# Could Subjective Cognitive Complaints Give the Facts of Objective Cognitive Impairments in Non‐Central Nervous System Cancer? A Systematic Review

**DOI:** 10.1002/brb3.71622

**Published:** 2026-08-30

**Authors:** Chieh‐Ning Li, Nai‐Wen Guo, Bei‐Yi Su

**Affiliations:** ^1^ Institute of Allied Health Sciences, College of Medicine National Cheng Kung University Tainan Taiwan; ^2^ Institute of Behavioral Medicine, College of Medicine National Cheng Kung University Tainan Taiwan; ^3^ Clinical Psychology Department Kaohsiung Municipal Kai‐Syuan Psychiatric Hospital Kaohsiung Taiwan; ^4^ Department of Psychology Chung Shan Medical University Taichung Taiwan

**Keywords:** cognitive impairments, cancer, neuropsychological test, self‐report

## Abstract

**Introduction:**

Non‐central nervous system (non‐CNS) cancer patients experienced cognitive impairment, both subjectively and objectively. However, they are only weakly correlated. Previous studies indicated that subjective cognitive complaints (SCCs) could be a distinct neural phenotype from objective neuropsychological function. To explore what underlies SCCs, we examined the relationship between SCCs and objective neuropsychological assessment in this systematic review.

**Methods:**

Following the PRISMA guidelines, we searched multiple databases from September 2000 to November 2024 for cross‐sectional studies that directly investigated SCCs and neuropsychological assessment, excluding those involving neurological conditions. Sixteen of the 202 articles met the criteria; the total sample included 2093. We used multilevel meta‐analysis in R to analyze 156 correlation coefficients and estimated the sampling error variance, within‐study variance, and between‐study variance from the analysis.

**Results:**

The correlations between SCCs and objective neuropsychological examinations were low but significant. When using cognitive domains, female ratio, and age disparity as variables, 57.18% of the variance was attributed to within‐study variance. As the female ratio increased, correlations between SCCs and executive function were inconsistent but consistent with attention and memory. When considering the relationship with age disparity, the trend would be corrected.

**Conclusions:**

Different cognitive domains may show distinct trends, suggesting that neuropsychological functions can be predicted by SCCs when controlling for specific conditions. Potential mechanisms include hormonal influences, age‐related biological and psychosocial changes, and differences in emotional vulnerability across the lifespan. The present study highlights the importance of carefully evaluating subjective cognitive complaints within evidence‐based decision‐making processes.

## Graphical abstract

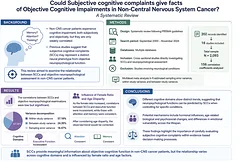

## 1 Background

The evidence indicated that non‐central nervous system (non‐CNS) cancer patients experienced cognitive impairments both subjectively and objectively (Hutterer and Oberndorfer 2021). Nevertheless, small correlations between the two were commonly reported by the studies (Hutchinson et al. 2012; Bray et al. 2018). Moreover, the subjective cognitive complaints (SCCs) were typically more severe than those observed in neuropsychological assessments (Henneghan et al. 2021). Many studies indicated that perceived cognitive impairments were associated with distress (O'Farrell et al. 2017). Henneghan et al. (2021) noted that distress and cognition may share similar neural prefrontal networks and intrinsic whole‐brain networks, suggesting that SCCs could be a distinct neural phenotype from the objective evidence.

Underlying SCCs, an accurate self‐report requires good self‐awareness, which is correlated with a paralimbic network including the medial prefrontal/anterior cingulate and other associated regions (Castro et al. 2023) and is associated with higher‐order cognitive functions, such as executive function and metacognition. It also requires effective management of information processing, including storage and retrieval, which are related to attention, memory, and other cognitive functions. Moreover, differences in the results of SCCs across different sexes and ages have always been mentioned (Knäuper et al. 2016; Asperholm et al. 2019).

To explore what was underlying SCCs, we examined the relationship between SCCs and objective neuropsychological assessment in cancer patients in this systematic review. In this review, we synthesize and analyze the reported correlations in the current literature. Furthermore, it has been studied that not only chemotherapy and other cancer treatments, but also multiple causes like genetic factors, biological factors, comorbidities, socio‐demographic variables, etc., were found to result in cognitive impairments in cancer patients (Fleming et al. 2023). In this review, several factors, including different cognitive domains, sex, and age, were selected to examine the relationships.

## 2 Methods

### 2.1 Data Sources, Search Strategy, and Selection

This systematic review adhered to the PRISMA statement (Page et al. 2021). The following academic databases were included in the search strategy: MEDLINE (OVID), Embase (Elsevier), Cochrane Library, CINAHL (EBSCOhost), Scopus, and Web of Science were employed to search for articles about SCCs and objective neuropsychological assessments in non‐CNS cancer patients. The publication year was not specified but was ultimately found to range from September 2000 to November 2024. The most recent search of these databases occurred on March 27, 2025 (Supplementary Material).

For a full search strategy for each database, broad search terms (Emtree, MeSH, and EBSCOhost) and synonyms were used (see the attached Supplementary Material). Terms relating to neoplasms, self‐report, neuropsychology, and cognitive dysfunction were included. To exclude patients with CNS cancer from the study, we conducted a literature search using the keyword “central nervous system neoplasms” and its synonyms, then subsequently excluded relevant publications. The eligibility criteria encompassed studies that address the SCCs and neuropsychological assessments of non‐CNS cancer patients. The searches conducted in each academic database used the following limits: (1) cancer patient, (2) English language; (3) Publications in peer‐reviewed academic journals or conference proceedings; (4) original research article; (5) cross‐sectional study design; and (6) adults (>18 years old). Publications from any year were considered for inclusion. The patient population required a diagnosis of cancer according to the TNM Staging System. Studies were eligible if they had a (i) cross‐sectional study design, (ii) examined the relationship between the results of SCCs and neuropsychological assessments, and (iii) had an exclusion to rule out patients with neurological problems. All titles, abstracts, and full texts of the studies obtained from the search strategy were independently screened by two authors, and disagreements were settled with the other author to ensure the studies' eligibility.

### 2.2 Data Extraction

The extracted data variables (See Table 1) include author and publication year, sample characteristics (Cancer type and sample size), female ratio, range of age, measures of SCCs and neuropsychological assessments, and correlations between SCCs and neuropsychological assessments.

**TABLE 1:** Characteristics of included studies.

Author and publication year	Sample characteristics (*n* = sample size)	Female ratio (%)	Range of Age	SCCs measures	Neuropsychological assessments measure	Correlations between SCCs and neuropsychological assessments
Rick et al. (2018)	Breast Cancer (*n =* 476)	100.00	24–62 (50.00 ± NR)	FEDA	NeuroCog	Positive correlations were weak (*r* < 0.3) but found for almost all parameters.
Poppelreuter et al. (2004)	Various types of cancer (*n =* 119)	67.23 (M = 39, F = 80)	20–75 (52.26 ± 12.30)	FEDA	WMS‐R, TMT, TAP, MWT‐B	**Attention (TAP)**: FEDA1: Tonic alertness (*r* = ‒0.07), phasic alertness (*r* = ‐0.01), alertness index (*r* = *‒*0.17), divided attention (*r* = 0.01), change of reaction (*r* = *‒*0.07); FEDA2: tonic alertness (*r* = *‒*0.03), phasic alertness (*r* = 0.01), alertness index (*r* = *‒*0.02), divided attention (*r* = 0.13), change of reaction (*r* = 0.00); FEDA3: Tonic alertness (*r* = 0.06), phasic alertness (*r* = 0.04), alertness index (*r* = *‒*0.05), divided attention (*r* = *‒*0.02), change of reaction (*r* = 0.10); **Cognitive speed/flexibility**: FEDA1: TMT A (*r* = 0.06), TMT B(*r* = 0.09); FEDA2: TMT A (*r* = 0.13), TMT B(*r* = 0.22**); FEDA3: TMT A (*r* = 0.14), TMT B (*r* = 0.22*); **Memory (WMS)**: FEDA1: Logical memory I (*r* = 0.24*), logical memory II (*r* = 0.15), digit span forward (*r* = 0.11), digit span backward (*r* = 0.11); FEDA2: logical memory I (*r* = 0.07), logical memory II (*r* = 0.12), digit span forward (*r* = 0.18*), digit span backward (*r* = 0.20*); FEDA3: Logical memory I (*r* = 0.19*), logical memory II (*r* = 0.17), digit span forward (*r* = 0.23*), digit span backward (*r* = 0.11) *p* < 0.05*, *p* < 0.01**
Oh (2017)	Various types of cancer (*n =* 175)	36.00 (M = 112, F = 63)	30*‒*77 (58.54 ± 9.80)	ECog	MMSE	**Significant correlated**: Ecog and MMSE (*r* = *‒*0.32, *p* < 0.01)
Jung & Visovatti (2017)	Papillary Thyroid Cancer (*n =* 90)	100.00	18+ (48.94 ± 10.57)	AFI	DST, COWA, TCFI scores	**No significant correlation**. AFI & TCFI scores (*r* = 0.07)
O'Farrell et al. (2017)	Breast Cancer (*n =* 60)	100.00	34*‒*65 (52.35 ± 7.93)	FACT‐cog	WAIS‐III, TMT A&B, DST, PASAT, CCCs, COWAT, HVLT‐R, BVMT‐R, objective cognitive summary score (COGSUM)	**No significant correlation**: The bivariate correlation between post‐treatment FACT‐Cog PCI and COGSUM scores (i.e., at T6) was nonsignificant (*r* = 0.056; *p* = 0.710).
Eggen et al. (2022)	metastatic non‐small cell lung cancer (mNSCLC) (*n =* 77)	49.35 (M = 39, F = 38)	37*‒*82 (62 ± NR)	FACT‐Cog	HVLT‐R, TMTA &B, COWAT	**No significant correlation**. Cognitive impairment and cognitive concerns were not directly correlated (Pearson *r* = −0.11; *p* = 0.93).
Vayyat et al. (2024)	Sarcoma patients (*n =* 90)	37.78 (M = 55, F = 35)	16*‒*40 (pre‐CT 33.4 ± 4.2; During CT 31 ± 3.8; post‐CT 35.7 ± 4.4)	FACT‐Cog	ACE‐III, Flanker's task, Sternberg's task, Face‐word Stroop task, Word‐face Stroop task	**Partial show significant correlation**: During Cx group FACT‐Cog Oth: attention domain of ACE‐III(*r* = *‒*0.53**) FACT‐Cog QoL: reaction times of Flanker's (*r* = 0.44*), Sternberg's tasks (*r* = 0.42*) Post Cx group FACT‐Cog Oth: total ACE‐III scores (*r* = 0.53**), memory domain of ACE‐III (*r* = 0.42*) *p* < 0.05*, *p* < 0.01**
McDowell et al. (2017)	NasoPharyngeal cancer patients (*n =* 103)	34.95 (M = 67, F = 36)	21*‒*77 (M = 56 ± NR)	FrSBe, MDASI (memory problems scale)	MoCA	**Significant correlated**: MoCA & MDASI (*r* = 0.26, *p* < 0.01).
Weis et al. (2009)	Breast Cancer patients (*n =* 90)	100.00	32*‒*63 (49.7 ± 7.6)	EORTC‐scale, FEDA	TAP, RBMT, WMS‐R, LGT‐3	**Significant correlated**: EORTC‐scale (two item about memory and attention deficits in general): Alertness tonic (*r* = 0.231*, *p* = 0.029), alertness phasic (*r* = 0.279**, *p* = 0.008), divided attention, reaction time(*r* = 0.105, *p* = 0.330), divided attention, sum of omissions and false reactions (*r* = *‒*0.162, *p* = 0.129), Go/No Go, reaction time (*r* = 0.178, *p* = 0.095); sustained attention, false reactions (*r* = *‒*0.258*, *p* = 0.015); RBMT ’story’ immediately (*r* = 0.236*, *p* = 0.026); RBMT ’story’ delayed (*r* = 0.293**, *p* = 0.005); LGT‐3 ’objects’ (*r* = 0.213*, *p* = 0.045). FEDA1: Alertness tonic (*r* = 0.247*, *p* = 0.020), alertness phasic (*r* = 0.318**, *p* = 0.003), divided attention, reaction time(*r* = 0.335**, *p* = 0.001), divided attention, sum of omissions and false reactions (*r* = *‒*0.213, *p* = 0.047), Go/No Go, reaction time (*r* = 0.250*, *p* = 0.019); sustained attention, false reactions (*r* = *‒*0.198, *p* = 0.064); RBMT ’story’ immediately (*r* = 0.109, *p* = 0.313); RBMT ’story’ delayed (*r* = 0.131, *p* = 0.224); LGT‐3 ’objects’ (*r* = 0.149, *p* = 0.165). *p* < 0.05*, *p* < 0.01**
Paquet et al. (2018)	Breast Cancer (*n =* 80)	100.00	30*‒*75 (54.10 ± 9.50)	PRMQ	MIST, WMS‐IV	**No significant correlation**. PM complaints: PM Mist (*r* = *‒*0.032), RM immediate (*r* = *‒*0.043), RM Delayed (*r* = 0.010), RM Recognition (*r* = 0.091); RM complaints: PM Mist (*r* = *‒*0.182), RM immediate (*r* = *‒*0.137), RM Delayed (*r* = *‒*0.118), RM Recognition (*r* = *‒*0.065). *p*<0.05*, *p*<0.01**
Hii et al. (2021)	Breast Cancer (*n =* 162)	100.00	18+ (55.00 ± 11.42)	PDQ‐5M	DSST	**No significant correlation**. DSST: Total PDQ (*r* = *‒*0.052), PDQ5(1) (*r* = 0.032), PDQ5(2) (*r* = *‒*0.016), PDQ5(3) (*r* = *‒*0.102*), PDQ5(4) (*r* = 0.102*), PDQ5(5) (*r* = 0.046). *p* < 0.05*, *p* < 0.01**
Kang et al. (2019)	non‐small‐cell lung cancer (NSCLC) (*n =* 113)	NR	NR (Untreated group: 59.05 ± 6.17; chemotherapy group: 58.10 ± 8.64 targeted therapy group: 57.82 ± 8.20)	FACT‐cog	MMSE, WAIS‐IV, CTT	**No significant correlation**. FACT‐cog (Untreated group): MMSE (*r* = 0.064), Vocabulary (*r* = *‒*0.055), DSS (*r* = *‒*0.255), Digit span (*r* = *‒*0.214), CTT1‐CT (*r* = *‒*0.255), CTT1‐error (*r* = *‒*0.092), CTT2‐CT (*r* = *‒*0.373*), CTT2 Number error (*r* = 0.217), CTT2 Color error (*r* = *‒*0.048). FACT‐cog (Chemotherapy group): MMSE (*r* = *‒*0.059), Vocabulary (*r* = *‒*0.152), DSS (*r* = *‒*0.146), Digit span (*r* = *‒*0.081), CTT1‐CT (*r* = *‒*0.057), CTT1‐error (*r* = 0.038), CTT2‐CT (*r* = 0.271), CTT2 Number error (*r* = *‒*0.361*), CTT2 Color error (*r* = 0.139). FACT‐cog (targeted therapy group): MMSE (*r* = *‒*0.023), Vocabulary (*r* = *‒*0.071), DSS (*r* = *‒*0.081), Digit span (*r* = *‒*0.014), CTT1‐CT (*r* = *‒*0.073), CTT1‐error (*r* = 0.199), CTT2‐CT (*r* = 0.067), CTT2 Number error (*r* = *‒*0.055), CTT2 Color error (*r* = *‒*0.023). *p* < 0.05*, *p* < 0.01**
Durán‐Gómez et al. (2022)	Breast Cancer (*n =* 180)	100.00	29*‒*83 (53.87 ± 10.54)	FACT‐Cog	SVF, PVF	**Significant correlated**: between all cognitive self‐report and NPT. FACT‐cog PCI: PVF(*r* = 0.516**), SVF(*r* = 0.630**) FACT‐cog Oth: PVF(*r* = 0.170*), SVF(*r* = 0.159*) FACT‐cog PCA: PVF(*r* = 0.675**), SVF(*r* = 0.648**) FACT‐cog QoL: PVF(*r* = 0.452**), SVF(*r* = 0.153*) *p* < 0.05*, *p* < 0.01**
Barton et al. (2013)	Breast Cancer (*n =* 210)	100.00	18+ (NR)	PHS (cognitive subscale)	HSCS, TMTA&B	**Partial show significant correlation**: (6 months follow‐up) PHS Stay focused: HSCS attention concentration (*r* = *‒*0.06), TMT A(*r* = 0.00), TMTB(*r* = *‒*0.02) PHS Think clearly: HSCS attention concentration (*r* = 0.04), TMT A(*r* = 0.20*), TMTB(*r* = 0.10) PHS Balance checkbook: HSCS attention concentration (*r* = 0.09), TMT A(*r* = 0.28*), TMTB(*r* = 0.21*) *p* = 0.02*
Boone et al. (2022)	Breast Cancer (*n =* 22)	100.00	18+ (53.60 ± 12.40)	FACT‐cog, CFQ	DPX	**Partial shows significant correlation**: FACT‐cog PCI: DPX Error rate AX(*r* = 0.10), DPX Error rate AY(*r* = 0.03), DPX Error rate BX(*r* = *‒*0.42), DPX Error rate BY(*r* = *‒*0.15), DPX Reaction Time AX(*r* = *‒*0.12), DPX Reaction Time AY(*r* = *‒*0.20), DPX Reaction Time BX(*r* = 0.09), DPX Reaction Time BY(*r* = *‒*0.07) FACT‐cog PCA: DPX Error rate AX(*r* = *‒*0.13), DPX Error rate AY(*r* = *‒*0.04), DPX Error rate BX(*r* = *‒*0.33), DPX Error rate BY(*r* = *‒*0.38), DPX Reaction Time AX(*r* = *‒*0.18), DPX Reaction Time AY(*r* = *‒*0.28), DPX Reaction Time BX(*r* = 0.00), DPX Reaction Time BY(*r* = *‒*0.18) CFQ: DPX Error rate AX(*r* = 0.10), DPX Error rate AY(*r* = 0.05), DPX Error rate BX(*r* = 0.44*), DPX Error rate BY(*r* = 0.20), DPX Reaction Time AX(*r* = *‒*0.17), DPX Reaction Time AY(*r* = *‒*0.14), DPX Reaction Time BX(*r* = *‒*0.24), DPX Reaction Time BY(*r* = *‒*0.20) *p* < 0.05*
LaLonde et al. (2021)	Hematopoietic stem cell transplant (HSCT) recipients (*n =* 46)	47.83 (Male 52%)	27–72 (56.00 ± 10.00)	FACT‐cog	CogState	**Partial shows significant correlation**: (100‐days follow‐up) FACT‐cog PCI: Psychomotor Speed (*r* = *‒*0.23), Attention (*r* = 0.39*), Visual Learning (*r* = 0.34*), Working Memory (*r* = 0.24), Problem Solving (*r* = *‒*0.24), Delayed Memory (*r* = *‒*0.22) FACT‐cog Oth: Psychomotor Speed (*r* = *‒*0.31), Attention (*r* = 0.44**), Visual Learning (*r* = 0.51***), Working Memory (*r* = 0.25), Problem Solving (*r* = *‒*0.18), Delayed Memory (*r* = *‒*0.22) *p* < 0.05*, *p* < 0.01**, *p* < 0.001***

Abbreviations: ACE = Addenbrooke Cognitive Examination, AFI = The Attentional Function Index, ANT = Attention Network Test, APM III = Raven's Advanced Progressive Matrices Test, BVMT‐R = Brief Visuospatial Memory Test—Revised, CCCS = Auditory Consonant Trigrams Test, CFQ = Cognitive Failures Questionnaire, COWAT = Controlled Oral Word Association Test, CPT = Continual Performance Task, CT = Chemotherapy Treatment, CTT = Color Trails Test, CVLT‐II = California Verbal Learning Test Second Edition, CWIT = Color‐Word Interference Test, DPX = Dot Pattern Expectancy, DSST = Digit Symbol Substitution Test, DST = Digit Span Test, ECOG = Everyday Cognition Scale, EMQ = Everyday Memory Questionnaire, FACT‐COG = Functional Assessment of Cancer Therapy Cognitive Function, FEDA = The Questionnaire on Experienced Deficits of Attention, HSCS = High Sensitivity Cognitive Screen, HVLT‐R = Hopkins Verbal Learning Test‐Revised, LGT‐3 = Lern‐ und Geda¨chtnistest (Learning and Memory‐Test), LNS = Letter Number Sequencing, LPS = Leistungs Pruef System, MIST = Memory for Intention Screening Test, MMSE = Mini Mental State Examination, MOCA = Montreal Cognitive Assessment, MOT = Multiple Object Tracking Task, MWT‐A = Mehrfachwah Wortschatz Intelligenz Test‐A, MWTB = Multiple‐Choice Vocabulary Test, NAB = Neuropsychological Assessment Battery, NART = National Adult Reading Test, NR = Not Reported, OFT = Orthographical Fluency Test, PASAT = Paced Auditory Serial Addition Task, PDQ5‐M = Perceived Deficit Questionnaire 5, PHQ‐9 = Patient Health Questionnaire‐9, PRMQ = Prospective and Retrospective Memory Questionnaire, PROMIS = Patient‐Reported Outcomes Measurement Information System, PVF = Phonological Verbal Fluency, RAVLT = Rey Auditory Verbal Learning Test, RBMT = Rivermead Behavioural Memory Test, ROCFT = The Rey–Osterrieth Complex Figure Test, RWT = Regensburger Word Fluency Test, SCWT = Stroop Color and Word Test, SFT = Semantic Fluency Test, SVF = Verbal Semantic Fluency, TAP = The Computer‐Based Test Battery for Assessment of Attention, TMT = Trail Making Test, VLMT = Verbal Learning Memory Test, WAIS‐IV = Wechsler Adult Intelligence Scale, Fourth Edition, WMS‐IV = Wechsler Memory Scale IV, WMS‐R = The Wechsler Memory Scale Revised.

### 2.3 Synthesis

For the meta‐correlation, we referred to the evidence in “Doing Meta‐Analysis in R: A Hands‐on Guide” (Harrer et al. 2021). We performed a multilevel meta‐analysis (MLMA), namely three‐level meta‐analysis models in R statistical computing software. In the model, the sampling error variance from all selected studies was at level 1, while variance not attributable to sampling error was at levels 2 and 3, indicating the within‐study variance and between‐study variance, respectively. Additionally, we examined publication bias using Egger's regression test, the Trim and Fill analysis, and a leave‐one‐out approach.

The dependent variable was the correlation coefficients between SCCs and neuropsychological assessments from the selected studies. Regarding the independent variables, we categorized neuropsychological assessments into various cognitive domains (see Table 2), including general cognitive function, attention, alertness, speed/psychomotor speed, memory, executive function, and others. Moreover, we defined the female ratio from the selected studies as the proportion of females in the sample. Instead of using the average age, we used the range of ages to represent the age of our population. A few studies did not provide the range, but we calculated four times the standard deviation from the average age as the value.

**TABLE 2:** Definition of the measures.

Cognitive domains	Measures of neuropsychological assessments
General cognitive function	NeuroCog, MMSE, TCFI, COGSUM, COG‐comp, ACE‐III, MoCA
Attention	TAP Divided attention, TAP Change of reaction, TMTA, attention domain of ACE‐III, TAP Divided attention (reaction time), TAP Divided attention (sum of omissions and false), TAP Go/No Go reaction time, TAP Sustained attention (false reactions), Digit Span, CTT1‐error, CTT2‐number error, CTT2‐color error, HSCS, DPX error rate BX, CogState‐Attention
Alertness	TAP Tonic alertness, TAP Phasic alertness, TAP Alertness index,
Speed/psychomotor speed	DSST, DSS, CTT1‐CT, CTT2‐CT, DPX reaction time BX, CogState‐Psychomotor Speed
Memory	WMS‐Logical memory I, WMS‐Logical memory II, Digit Span forward, Digit Span backward, memory domain of ACE‐III, RBMT'story'delayed’, RBMT'story'immediately’, LGT‐3'objects', PM Mist, RM immediate, RM delayed, RM Recognition, CogState‐Visual Learning, CogState‐Delayed Memory,
Executive function	TMTB, reaction times of Flanker's, Sternberg's tasks, phonological verbal fluency, verbal semantic fluency test, DPX Reaction time AY, CogState‐WM, CogState‐Problem Sovling
Others	Vocabulary, DPX Error rate AX, DPX Error rate AY, DPX Error rate BY, DPX Reaction Time AX, DPX Reaction Time BY,

## 3 Results

### 3.1 Study selection

**FIGURE 1:**
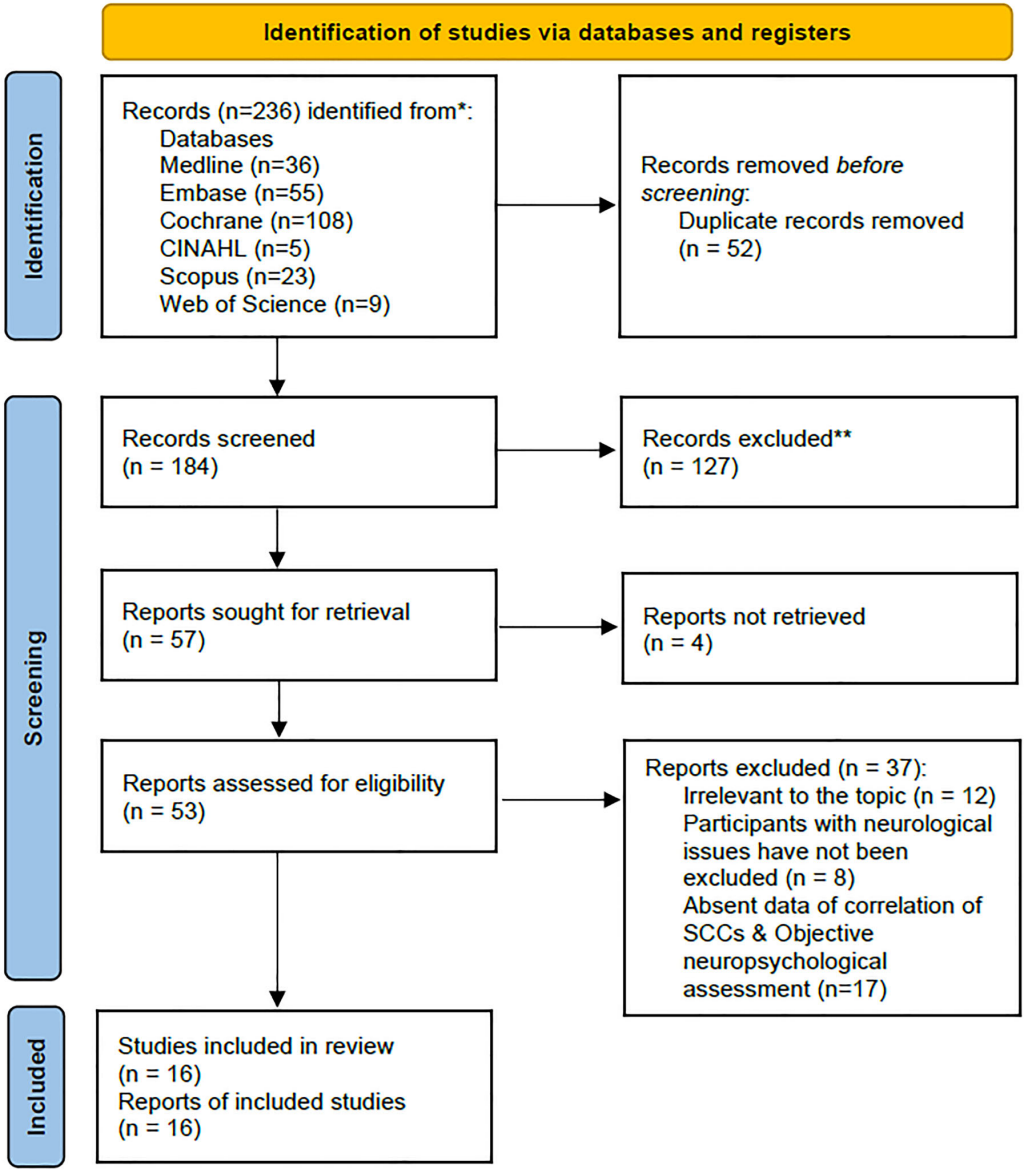
PRISMA flow diagram.

Two hundred and thirty‐six eligible studies were identified through a search of research databases. After removing duplicates (*n =* 52), 184 studies remained. Following the screening process, 16 studies were included for final analysis. The selection process is illustrated in Figure 1 of the PRISMA flow diagram.

### 3.2 Study Characteristics

This study investigates the association between SCCs and neuropsychological assessments. A cross‐sectional approach was employed to search for relevant research. Sixteen studies met the criteria and were included in the final analysis. Sample sizes ranged from 22 to 476, ranging from 18 to 83 years. The total number of cancer patients included in all studies for this research was 2093, comprising 1280 individuals with breast cancer. The sample size for females after excluding breast cancer was 363, while the sample size for males was 337. There were 113 individuals whose sex was unspecified.

Eight (Weis et al. 2009; Barton et al. 2013; O'Farrell et al. 2017; Paquet et al. 2018; Rick et al. 2018; Hii et al. 2021; Boone et al. 2022; Durán‐Gómez et al. 2022) of 16 studies investigated breast cancer patients with a total population of 1280. Two studies (Kang et al. 2019; Eggen et al. 2022) investigated non‐small cell lung cancer, with a population of 190. One study (Jung and Vi‐sovatti 2017) investigated papillary thyroid cancer, with a population of 90. One study (LaLonde et al. 2021) investigated hematopoietic stem cell transplant recipients, with a population of 24. One study (Vayyat et al. 2024) investigated sarcoma, with a population of 90. One study (McDowell et al. 2017) investigated nasopharyngeal cancer, with a population of 103. Two studies (Poppelreuter et al. 2004; Oh 2017) investigated two or more types of cancer in a population of 294.

Cases were recruited at various time points, including before cancer treatment, after treatment, and with follow‐up periods extending up to six years post‐treatment. The cancer patients included in the study might undergo various types of cancer treatments, including surgery, chemotherapy, targeted therapy, hormone therapy, and radiation therapy.

### 3.3 Meta‐Analysis of Correlation Between SCCs and Neuropsychological Measures

The total amounts extracted were 156 from sixteen studies that were included. estimate of our pooled effect, which was *z* =  0.1142 (95%CI: 0.0273–0.2010, *p* = 0.0103), transform the effect back to a normal correlation, which was *r* = 0.1137 (95%CI: 0.0273–0.1984).

**FIGURE 2:**
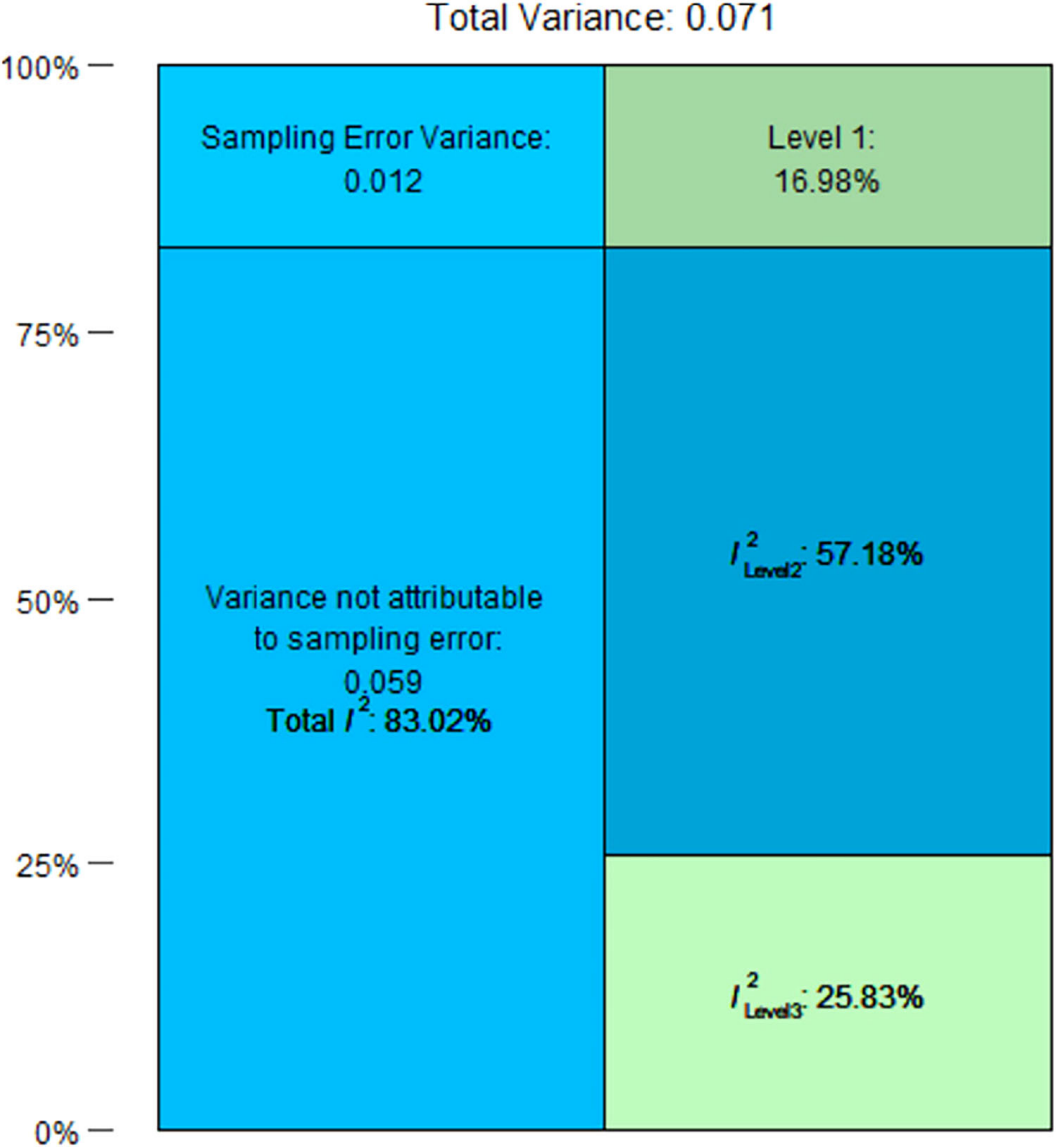
Distribution of variance across levels.

The variance analysis revealed that Level 1 (sampling error variance) accounted for $I^{2}$= 16.98%, Level 2 (within‐cluster variance) for $I^{2}$= 57.18%, and Level 3 (between‐study variance) for $I^{2}$= 25.83%, indicating the main variance was within the study. (Shown in Figure 2)

#### 3.3.1 Publication Bias and Sensitivity Analysis at the Between‐Study Level

**FIGURE 3:**
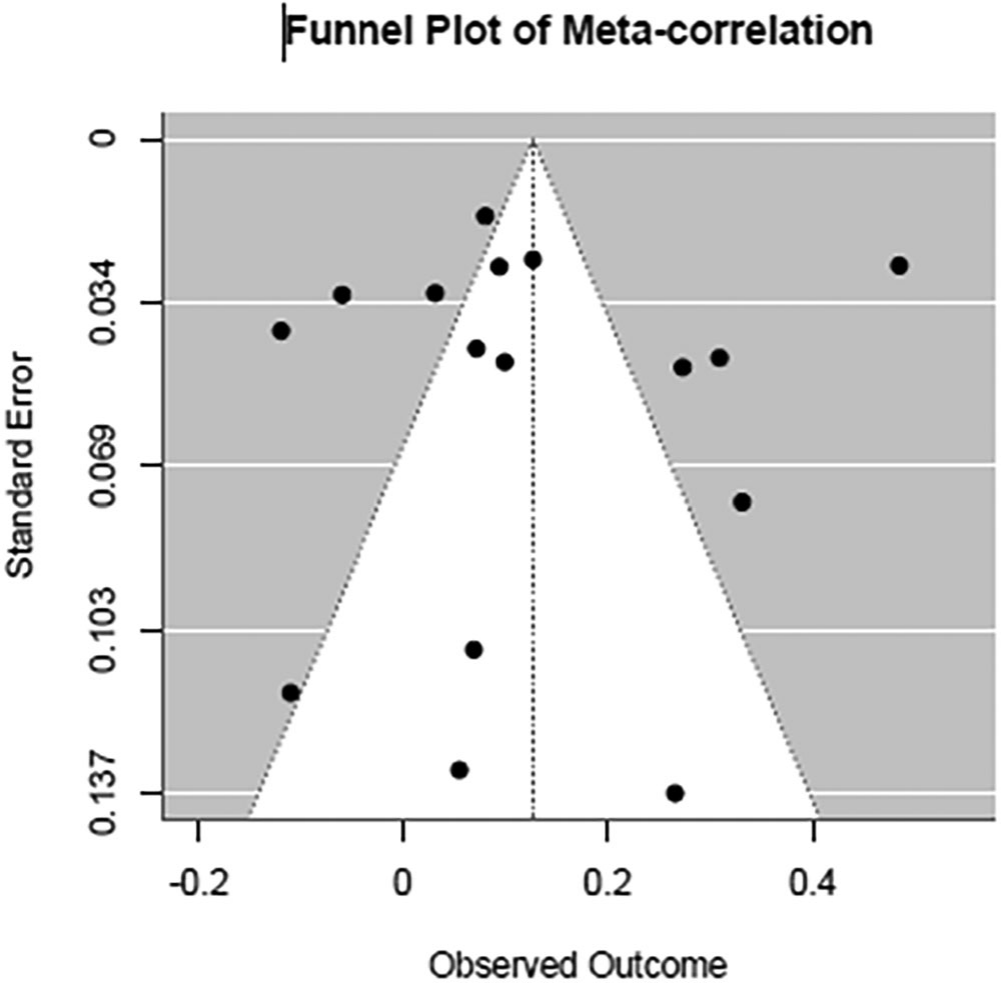
Funnel plot.

We examined publication bias using Egger's regression test, trim and fill analysis, and a leave‐one‐out approach on the selected studies. For one effect size with one study, we used inverse‐variance weighted pooling to integrate multiple effect size coefficients into a single effect size. The results were in Table 3 and Figure 3 (funnel plot).

**TABLE 3:** Publication bias and sensitivity analysis.

Test		z	*p*‐value	Limit estimate/estimate	CI
Egger's regression		‒0.1952	0.8453	0.1404	[‒0.0153, 0.2960]
Trim‐and‐Fill		3.3312	0.0009	0.1454	[0.0594, 0.2315]
Leave‐One‐Out					
	‒1	2.5955	0.0094	0.1145	[0.0280, 0.2010]
	‒2	2.8363	0.0046	0.1310	[0.0405, 0.2216]
	‒3	2.6187	0.0088	0.1148	[0.0289, 0.2007]
	‒4	2.8852	0.0039	0.1306	[0.0419, 0.2192]
	‒5	2.9118	0.0036	0.1308	[0.0427, 0.2188]
	‒6	3.2002	0.0014	0.1394	[0.0540, 0.2247]
	‒7	2.6117	0.0090	0.1171	[0.0292, 0.2051]
	‒8	2.7257	0.0064	0.1214	[0.0341, 0.2088]
	‒9	2.7524	0.0059	0.1274	[0.0367, 0.2182]
	‒10	3.4674	0.0005	0.1460	[0.0635, 0.2285]
	‒11	2.9484	0.0032	0.1346	[0.0451, 0.2241]
	‒12	3.2319	0.0012	0.1417	[0.0558, 0.2276]
	‒13	2.7702	0.0056	0.0996	[0.0291, 0.1702]
	‒14	2.8110	0.0049	0.1299	[0.0393, 0.2205]
	‒15	2.8087	0.0050	0.1294	[0.0391, 0.2197]
	‒16	2.8593	0.0042	0.1314	[0.0413, 0.2215]

Among the studies, there was no evident bias as indicated by the Egger's regression test and the funnel plot analysis; however, the trim‐and‐fill analysis suggested a possible lack of evidence on the right side. However, after the correction, it still showed positive significance (*z* = 3.31, *p* < 0.001, *r* ≈ 0.14, 95% CI [0.06, 0.23]), which demonstrates that the bias was limited to the result. In the Leave‐one‐out analysis, regardless of which study was removed, overall correlations remained around *r* = 0.10–0.15, without a zero crossing of the confidence interval, indicating stability of the main effect.

#### 3.3.2 Moderator Effects at the Within‐Study Level

As we used the female ratio, the age range, and cognitive domains (executive function as the reference group, which is the basis for accurate self‐report) as the moderators of the MLMA, the multivariate meta‐analysis model was significant with F (df1 = 24, df2 = 131) = 2.8932, *p* < 0.0001. Interactions were shown between moderators.

Table 4 demonstrated our multilevel meta‐analysis models predicting the correlation between SCCs and neuropsychological assessments. It showed that among several cognitive domains, different correlation trends depend on the female ratio, the age range, and their interactions. We could easily identify different correlation patterns in various cognitive domains, such as attention, alertness, and memory, which were significantly distinct from those of executive function. We observed that when considering the female ratio or the age range separately in these cognitive domains, the correlations increased; however, when considering both, the correlation was mildly corrected. On the contrary, in the executive function and other domains, which were not significantly different from it, when considering the female ratio or the age range, respectively, the correlations decreased; however, when considering both, the correlation was either mildly corrected. Although the trend of correlations may differ, we can observe the effect with the interaction between the female ratio and the age range in the population.

**TABLE 4:** Multivariate meta‐analysis predicting the correlation between cognitive self‐report and neuropsychological assessment.

Predictor	Estimate(β)	SE/ 95% CI	t (df = 131)	*p*
Intercept (executive function)	2.47	0.75[0.98, 3.96]	3.29	0.0013**
Female ratio	−4.07	1.46[‐6.96, ‒1.18]	−2.79	0.0061**
Age difference	−0.06	0.02[‐0.09, ‒0.02]	‒3.29	0.0013**
Cognitive domains (vs. executive function)				
General cognitive function	‒1.85	1.16[‒4.15, 0.46]	‒1.59	0.1152
Attention	‒3.12	0.88[‒4.87, ‒1.37]	‒3.53	0.0006***
Alertness	‒1.93	0.84[‒3.59, ‒0.26]	‒2.29	0.0237*
Speed/psychomotor speed	‒4.67	2.51[‒9.64, 0.29]	‒1.86	0.0649.
Memory	‒2.55	1.06[‒4.64, ‒0.46]	‒2.42	0.0171*
Others	‒0.30	0.57[‒1.43, 0.84]	‒0.51	0.6081
**Interaction**				
Female ratio X general cognitive function	3.39	2.33[‒1.23, 8.00]	1.45	0.1000
Female ratio X Attention	4.67	1.60[1.51, 7.82]	2.92	0.0041**
Female ratio X alertness	2.61	1.17[0.29, 4.93]	2.23	0.0275*
Female ratio X speed/psychomotor speed	8.01	4.82[‒1.53, 17.54]	1.66	0.0992.
Female ratio X memory	4.80	1.74[1.36, 8.24]	2.76	0.0066**
Female ratio X others	0.01	1.37[‒2.69, 2.71]	0.01	0.9951
Female ratio X age difference	0.09	0.03[0.04, 0.15]	3.17	0.0019**
Age difference X general cognitive function	0.05	0.03[‒0.01, 0.10]	1.66	0.1000
Age difference X attention	0.07	0.02[0.03, 0.11]	3.53	0.0006***
Age difference X alertness	—	—	—	—
Age difference X speed/psychomotor speed	0.10	0.06[‒0.01, 0.21]	1.75	0.821
Age difference X memory	0.07	0.02[0.02, 0.12]	2.95	0.0038**
Age difference X others	0.00	0.03[‒0.07, 007]	0.03	0.9797
Female ratio X age difference X general cognitive function	‒0.08	0.06[‒0.19, 0.03]	‒1.43	0.1544
Female ratio X age difference X attention	‒0.11	0.03[‒0.17, ‒0.04]	‒3.21	0.0017**
Female ratio X age difference X alertness	—	—	—	—
Female ratio X age difference X speed/psychomotor speed	‒0.17	0.10[‒0.38, 0.03]	‒1.67	0.0969.
Female ratio X Age difference X memory	‒0.13	0.04[‒0.20, ‒0.05]	‒3.33	0.0011**
Female ratio X age difference X others	—	—	—	—

^a^ *p* < 0.05^*^, *p* < 0.01^**^, *p* < 0.001^***.^

## 4 Discussion

Previous studies prevalently mentioned that the correlation between SCCs and neuropsychological assessments could be weak or absent, while the two assessed different aspects (Pace et al. 2024). In our results, we found a small but significant relationship that was not negligible. Most variance from correlation coefficients was not between the studies but rather within each study. And we found that sex, age, and cognitive domains together could explain the within‐variance.

Different cognitive domains, such as attention, memory, and executive function, showed correlations with varying trends. It was pervasively accepted that cancer‐related cognitive impairment was diffuse and relatively nonspecific (Vardy et al. 2008; Sheppard et al. 2024). However, a recent review (Nakamura et al. 2024) examining the effects of cognitive rehabilitation on adult cancer survivors also noted differences across cognitive domains. A systematic review of SCCs using neuropsychological tools (Webster‐Cordero and Giménez‐Llort 2025) found that, despite high heterogeneity among SCCs, the cognitive domains they encompass include executive function, language, and memory, suggesting that SCCs correlate with specific cognitive domains. To our evidence, an increase or decrease in the consistency was observed across different cognitive domains, accompanied by an elevated female proportion and age disparity in the cancer population, suggesting that neuropsychological functions can be predicted by SCCs when controlling for specific conditions.

Regarding the sex effect, in this review, the male population was small; nevertheless, we found that when the female ratio increased, the consistency between SCCs and objective neuropsychological assessments varied among cancer patients. Mayo et al. (2024) used cancer patients seeking psychosocial support as their population and found that over half of them reported cognitive symptoms of any severity. Moreover, the raised risks of moderate to severe cognitive symptoms were among their female cancer patients, including not only breast cancer but also hematological cancer.

In our results, as the female population increased, SCCs were consistent with attention and memory, but not with executive function performance. The results implied that the more severe SCCs the female cancer patients report, the more significant the deficits of their attention and memory function should be noted. However, the more severe SCCs the female cancer patients reported, the better the objective executive function was. It was confirmed that SCCs were associated with executive function (Webster‐Cordero and Giménez‐Llort 2022). However, in our results, as the female ratio of cancer patients increased, the trend went in the opposite direction, suggesting that other moderators should be considered.

The perspective on hot and cold executive function suggests that SCCs should consider the emotion or distress associated with hot executive function. However, most neuropsychological assessments were for cold executive function. This perspective was also mentioned in Van Patten et al.’s (2025) recent work on a transdiagnostic perspective. Moreover, referring to the transdiagnostic perspective, attention and memory would be underlying functions when discussing affective problems like depression and anxiety, implying close relationships.

Emotional factors and adjustment issues were mentioned on SCCs before; in the cancer population, a clear sex effect was also confirmed (Webster‐Cordero and Giménez‐Llort 2022). Although depression and anxiety were not included as moderator variables in the present analysis due to the limited availability of comparable data across studies, these factors were frequently discussed as potential explanations for the inconsistency between SCCs and neuropsychological assessment. Indeed, most studies included in the present review reported significant associations between SCCs and symptoms of depression and anxiety (Poppelreuter et al. 2004; Weis et al. 2009; Oh 2017; O'Farrell et al. 2017; Paquet et al. 2018; Rick et al. 2018; Kang et al. 2019; LaLonde et al. 2021; Hii et al. 2021; Eggen et al. 2022). Previous research has consistently demonstrated a higher prevalence of depression and anxiety among women across different stages of life (Farhane‐Medina et al. 2022; Barry and Zdanys 2023). However, the findings of the present study suggest that not only sex, but also age, may influence the relationship between SCCs and neuropsychological assessment. This observation is consistent with previous literature indicating that the combined effects of sex and age should be considered within both biological and psychosocial frameworks (Faravelli et al. 2013; Vasiliadis et al. 2020; Farhane‐Medina et al. 2022). Potential mechanisms may include hormonal influences, age‐related biological and psychosocial changes, and differences in emotional vulnerability across the lifespan. By identifying both sex and age as significant moderators, the present study highlights the importance of considering broader contextual factors when interpreting the inconsistency between SCCs and neuropsychological assessment in patients with cancer.

Mayo et al. (2024) mentioned the menopause induced by treatment or the toxic treatment effects from sex differences. Moreover, cognitive changes in female cancer patients vary with age, and the potential influences of hormones, menopause, pre‐treatment menopause, post‐treatment menopause, and hormone therapy should not be overlooked (Mandelblatt et al. 2013; Wefel et al. 2014). Previous non‐cancer research indicates that estrogen affects the hippocampus and other brain regions, as well as cognitive function (Schwabe et al. 2020; Coenjaerts et al. 2022; Nerattini et al. 2023; Julian et al. 2025). Different stages of female development, along with the influence of menstruation and hormones, pose varying risks of impaired subjective and objective memory (Li et al. 2022). Studies on cancer patients (Jenkins et al. 2007; Haggstrom et al. 2022) have shown that estrogen receptors are present in brain regions related to cognitive function, such as the prefrontal cortex, hippocampus and related cortex, and amygdala, indicating that endocrine therapy has a significant impact on cognitive function in breast cancer patients. Therefore, our results supported the previous perspective on the effects of endocrine and hormonal factors on cognitive function, and we noticed that these factors should not be excluded when discussing this issue.

### 4.1 Study Limitations and Future Directions

Due to limitations in including data on the correlation between SCCs and objective neuropsychological tests, only a small number of studies could be selected. This also limited the diversity of cancer types included in the studies, and the proportion of male samples was significantly lower than that of female samples, thus limiting the scope of the research results. Due to the limited data, depression, anxiety, and other psychosocial factors were not included as moderator variables in the present analysis. We would recommend further exploration by including these factors in future studies.

Given that sex and age may contribute to differences in the association between subjective and neuropsychological assessment, considering that the incidence of various cancer types differs across sex and age groups, future research should focus on specific cancer populations once a sufficient body of empirical evidence has accumulated. Such studies may help clarify the roles of particular psychosocial and biological factors underlying this relationship.

In addition, the present analysis was based primarily on cross‐sectional studies and included patients across all cancer stages. Therefore, it did not specifically address potential variations associated with changes over time or disease severity. Future studies are encouraged to focus on specific cancer types or treatment stages or to employ longitudinal designs to better understand how the relationship between SCCs and objective cognitive impairment may change over the course of the disease trajectory and treatment process.

### 4.2 Clinical Implications

Webster‐Cordero and Giménez‐Llort (2022) suggest that patients’ and/or informants’ complaint reports should not be underestimated, suggesting the value of self‐reports in the clinical setting. Although the correlation between SCCs and objective neuropsychological function was low, we still believe in their value, and our results showed signs of cognitive impairment by examining the consistency of the relationship across cognitive domains. Different sex and ages may exhibit distinct trends in relationships, suggesting that neuropsychological functions can be predicted by SCCs when controlling for specific conditions, and SCCs are not merely related to distress.

Furthermore, patients who report cognitive symptoms or emotional distress are frequently excluded from treatment protocols and clinical trials. However, the severity of SCCs may not accurately reflect objectively assessed cognitive functioning. As a result, relying solely on self‐report or the psychiatric diagnosis given when determining eligibility may introduce unintended bias (Dahò et al. 2025). The findings of the present study highlight the importance of carefully evaluating SCCs within evidence‐based decision‐making processes, thereby helping to ensure fair access to treatment, research participation, and other potentially beneficial opportunities, while minimizing the risk of ethically unjustified exclusion.

## 5 Conclusion

The correlation between SCCs and objective cognitive abilities is low but significant and remains meaningful to explore. Although more evidence is needed, the results suggest that neuropsychological functions can be predicted by SCCs when specific conditions are controlled for. The relationships show different trends by sex and ages, suggesting potential mechanisms may include hormonal influences, age‐related biological and psychosocial changes, and differences in emotional vulnerability across the lifespan, which further exploration is needed for. The present study also highlights the importance of carefully evaluating SCCs within evidence‐based decision‐making processes rather than relying solely on self‐report or the psychiatric diagnosis given.

## Author Contributions

**Nai‐Wen Guo**: methodology, validation, conceptualization, supervision, writing – review and editing, investigation, data curation. **Chieh‐Ning Li**: conceptualization, methodology, software, data curation, formal analysis, validation, investigation, visualization, project administration, writing – review and editing, writing – original draft. **Bei‐Yi Su**: methodology, data curation, validation.

## Funding

The authors have nothing to report.

## Ethics Statement

We carefully comply with all ethical standards when conducting this systematic review.

## Conflicts of Interest

The authors declare no conflicts of interest.

## Supporting information




**Supplementary Materials**: brb371622‐sup‐0001‐SuppMat.docx

## Data Availability

The authors confirm that the data supporting the findings of this study is available within the article or its supplementary materials.
