# Supplementary material for: Could Subjective Cognitive Complaints Give the Facts of Objective Cognitive Impairments in Non‐Central Nervous System Cancer? A Systematic Review
Source: Brain Behav. 2026 Aug 30;16(9):e71622. doi: 10.1002/brb3.71622 (PMC13527419; doi:10.1002/brb3.71622)
Supplement: Supplementary file 1 [file BRB3-16-e71622-s001.docx]

**Supplementary Material** – Search Strategy

**Key Words**

- Cancer OR
- Malignancy OR
- neoplasms OR
- oncology OR
- tumor

NOT

- central nervous system neoplasm OR
- CNS neoplasm OR
- central nervous system tumor OR
- CNS tumor

AND

- Subjective cognitive complaints OR
- subjective cognitive decline OR
- subjective memory complaints OR
- subjective memory decline OR
- perceived cognitive problems OR
- Self Report

AND

- Neuropsychology OR
- functioning of the brain and cognitive processes or behavior OR
- Neurocognitive OR
- Neuropsychological Tests

AND

- cognitive disorder OR
- cognitive defects OR
- cognitive deficits OR
- cognitive disability OR
- cognitive dysfunction OR
- cognitive impairments OR
- cognitive decline OR
- Mild Cognitive Impairment OR
- chemotherapy-related cognitive impairment OR
- cancer related cognitive impairment OR
- neurobehavioral Manifestations

AND

- Cross-Sectional Studies OR
- Cross section

**Criteria**

1. English language
2. Publications in a peer-reviewed academic journals or conference proceedings
3. original research article
4. cross-sectional study design
5. human subjects

**Databases**

1. MEDLINE(OVID)
2. Embase (Elsevier)
3. Cochrane Library
4. CINAHL(EBSCOhost)
5. Scopus
6. Web of Science

**Imports**

1. MEDLINE(OVID): 31 selected
2. Embase (Elsevier): 50 selected
3. Cochrane Library: 80 selected
4. CINAHL(EBSCOhost): 5 selected
5. Scopus: 22 selected
6. Web of Science: 14 selected
7. Total Import: **202 selected**, 50 duplicates removed, 152 total

| Database | # | Search syntax | Citations found |
| --- | --- | --- | --- |
| 1)   **Embase (Elsevier)** | 1 | (Cancer* OR malignanc* OR neoplas* OR oncologi* OR tumo*):ti,ab | 5549098 |
|  | 2 | " malignant neoplasm "/exp | **4954964** |
|  | 3 | ("central nervous system neoplasm*" OR " central nervous system tumo*" OR " CNS tumo*" OR "Central Nervous System Cancer" OR " cns neoplasm*" OR "central nervous system neoplasms, primary" OR " neoplasm*, cns" OR "neoplasms, central nervous system" OR " primary central nervous system neoplasm*" OR " tumors, central nervous system " ):ti,ab | **13972** |
|  | 4 | "central nervous system tumor"/exp | **485891** |
|  | 5 | (Subjective OR complaint* OR "subjective cognitive complaints" OR "subjective cognitive decline" OR "subjective memory complaints" OR "subjective memory decline" OR "perceived cognitive problems" OR "Report*, Self" OR "Self Report*" OR "Cognitive Symptom*"):ti,ab | **703948** |
|  | 6 | " self report "/exp | **170789** |
|  | 7 | (Neuropsycholog* OR "functioning of the brain and cognitive processes or behavior" OR "neuro-psychologi*" OR Neurocogniti*):ti,ab | **142539** |
|  | 8 | " Neuropsychology "/exp OR " neuropsychological assessment "/exp | **105417** |
|  | 9 | ("Cogniti* disorder*" OR "cogniti* defect*" OR "cogniti* deficit*" OR "cogniti* disability*" OR "cogniti* dysfunction*" OR "cogniti* impairment" OR "Cogniti* Decline*" OR "Mild Cognitive Impairment" OR " chemotherapy-related cognitive impairment " OR " cancer related cognitive impairment "):ti,ab | 222416 |
|  | 10 | " cognitive defect "/exp | **693223** |
|  | 11 | (#1 OR #2) NOT (#3 OR #4) AND (#5 OR #6) AND (#7 OR #8) AND (#9 OR #10) AND [embase]/lim | **449** |
|  | 12 | #11 AND ('cross-sectional study'/de OR 'correlational study'/de OR 'cross section*':ti,ab,kw OR crosssection*:ti,ab,kw OR crossection*:ti,ab,kw OR 'descriptive stud*':ti,ab,kw) | **55** |
| 2)   **MEDLINE (Ovid)** | 1 | (Cancer* OR malignanc* OR neoplas* OR oncologi* OR tumo*).ti,ab | 3974995 |
|  | 2 | exp " Neoplasms "/ | **4089833** |
|  | 3 | ("central nervous system neoplasm*" OR " central nervous system tumo*" OR " CNS tumo*" OR "Central Nervous System Cancer" OR " cns neoplasm*" OR "central nervous system neoplasms, primary" OR " neoplasm*, cns" OR "neoplasms, central nervous system" OR " primary central nervous system neoplasm*" OR " tumors, central nervous system ").ti,ab | **8383** |
|  | 4 | exp "Central Nervous System Neoplasms"/ | **220075** |
|  | 5 | (Subjective OR complaint* OR "subjective cognitive complaints" OR "subjective cognitive decline" OR "subjective memory complaints" OR "subjective memory decline" OR "perceived cognitive problems" OR "Report*, Self" OR "Self Report*" OR "Cognitive Symptom*").ti,ab | **509685** |
|  | 6 | exp " Self Report "/ | **47839** |
|  | 7 | (Neuropsycholog* OR "functioning of the brain and cognitive processes or behavior" OR "neuro-psychologi*" OR Neurocogniti*).ti,ab | **95765** |
|  | 8 | exp " Neuropsychology "/ or exp " Neuropsychological Tests "/ | **201456** |
|  | 9 | ("Cogniti* disorder*" OR "cogniti* defect*" OR "cogniti* deficit*" OR "cogniti* disability*" OR "cogniti* dysfunction*" OR "cogniti* impairment" OR "Cogniti* Decline*" OR "Mild Cognitive Impairment" OR " chemotherapy-related cognitive impairment " OR " cancer related cognitive impairment ").ti,ab | **150953** |
|  | 10 | exp " Cognitive Dysfunction "/ or exp " Chemotherapy-Related Cognitive Impairment "/ or exp " Neurobehavioral Manifestations "/ | **364534** |
|  | 11 | (1 OR 2) NOT (3 OR 4) AND (5 OR 6) AND (7 OR 8) AND (9 OR 10) | 289 |
|  | 12 | 11 AND (Cross-Sectional Studies/ OR ("cross section*" OR crosssection* OR crossection*).ti,ab.) | **36** |
| 3)   **Cochrane CENTRAL** | 1 | (Cancer* OR malignanc* OR neoplas* OR oncologi* OR tumo*):ti,ab | **242605** |
|  | 2 | [mh "Neoplasms"] | **126239** |
|  | 3 | (central nervous system neoplasm*) OR (central nervous system tumo*) OR (CNS tumo*) OR (Central Nervous System Cancer) OR (cns neoplasm*) OR (central nervous system neoplasms, primary) OR (neoplasm*, cns) OR (neoplasms, central nervous system) OR (primary central nervous system neoplasm*) OR (tumors, central nervous system):ti,ab | **5417** |
|  | 4 | [mh "Central Nervous System Neoplasms"] | **3594** |
|  | 5 | (Subjective OR complaint*) OR (subjective cognitive complaints) OR (subjective cognitive decline) OR (subjective memory complaints) OR (subjective memory decline) OR (perceived cognitive problems) OR (Report*, Self) OR (Self Report*) OR (Cognitive Symptom*):ti,ab | **147221** |
|  | 6 | [mh "Self Report"] | **3684** |
|  | 7 | Neuropsycholog* OR (functioning of the brain and cognitive processes or behavior) OR (neuro-psychologi*) OR (Neurocogniti*):ti,ab | 136130 |
|  | 8 | [mh "Neuropsychology"] or [mh "Neuropsychological Tests"] | **19951** |
|  | 9 | (Cogniti* disorder*) OR (cogniti* defect*) OR (cogniti* deficit*) OR (cogniti* disability*) OR (cogniti* dysfunction*) OR (cogniti* impairment) OR (Cogniti* Decline*) OR (Mild Cognitive Impairment) OR (chemotherapy-related cognitive impairment) OR (cancer related cognitive impairment):ti,ab | **71653** |
|  | 10 | [mh "Cognitive Dysfunction"] or [mh "Chemotherapy-Related Cognitive Impairment"] or [mh "Neurobehavioral Manifestations"] | **16292** |
|  | 11 | (#1 OR #2) NOT (#3 OR #4) AND (#5 OR #6) AND (#7 OR #8) AND (#9 OR #10) | **493** |
|  | 12 | #11 AND (((TI (systematic* n3 review*)) or (AB (systematic* n3 review*)) or (TI (systematic* n3 bibliographic*)) or (AB (systematic* n3 bibliographic*)) or (TI (systematic* n3 literature)) or (AB (systematic* n3 literature)) or (TI (comprehensive* n3 literature)) or (AB (comprehensive* n3 literature)) or (TI (comprehensive* n3 bibliographic*)) or (AB (comprehensive* n3 bibliographic*)) or (TI (integrative n3 review)) or (AB (integrative n3 review)) or (JN “Cochrane Database of Systematic Reviews”) or (TI (information n2 synthesis)) or (TI (data n2 synthesis)) or (AB (information n2 synthesis)) or (AB (data n2 synthesis)) or (TI (data n2 extract*)) or (AB (data n2 extract*)) or (TI (medline or pubmed or psyclit or cinahl or (psycinfo not “psycinfo database”) or “web of science” or scopus or embase)) or (AB (medline or pubmed or psyclit or cinahl or (psycinfo not “psycinfo database”) or “web of science” or scopus or embase)) or (MH “Systematic Review”) or (MH “Meta Analysis”) or (TI (meta-analy* or metaanaly*)) or (AB (meta-analy* or metaanaly*)))) | **108** |
| 4)   **CINAHL (EBSCOhost)** | 1 | ti (Cancer*) or ab (Cancer*) or ti (malignanc*) or ab (malignanc*) or ti (neoplas*) or ab (neoplas*) or ti (oncologi*) or ab (oncologi*) or ti (tumo*) or ab (tumo*) | **468722** |
|  | 2 | mh ("Neoplasms") | **95490** |
|  | 3 | ti ("central nervous system neoplasm*") or ab ("central nervous system neoplasm*") or ti ("central nervous system tumo*") or ab ("central nervous system tumo*") or ti ("CNS tumo*") or ab ("CNS tumo*") or ti ("Central Nervous System Cancer") or ab ("Central Nervous System Cancer") or ti ("cns neoplasm*") or ab ("cns neoplasm*") or ti ("central nervous system neoplasms, primary") or ab ("central nervous system neoplasms, primary") or ti ("neoplasm*, cns") or ab ("neoplasm*, cns") or ti ("neoplasms, central nervous system") or ab ("neoplasms, central nervous system") or ti ("primary central nervous system neoplasm*") or ab ("primary central nervous system neoplasm*") or ti ("tumors, central nervous system") or ab ("tumors, central nervous system") | **419** |
|  | 4 | mh ("Central Nervous System Neoplasms") | **2094** |
|  | 5 | ti ("Subjective OR complaint*") or ab ("Subjective OR complaint*") or ti ("subjective cognitive complaints") or ab ("subjective cognitive complaints") or ti ("subjective cognitive decline") or ab ("subjective cognitive decline") or ti ("subjective memory complaints") or ab ("subjective memory complaints") or ti ("subjective memory decline") or ab ("subjective memory decline") or ti ("perceived cognitive problems") or ab ("perceived cognitive problems") or ti ("Report*, Self") or ab ("Report*, Self") or ti ("Self Report*") or ab ("Self Report*") or ti ("Cognitive Symptom*") or ab ("Cognitive Symptom*") | **14713** |
|  | 6 | mh ("self report") | **83099** |
|  | 7 | ti (Neuropsycholog*) or ab (Neuropsycholog*) or ti ("functioning of the brain and cognitive processes or behavior") or ab ("functioning of the brain and cognitive processes or behavior") or ti ("neuro-psychologi*") or ab ("neuro-psychologi*") or ti ("Neurocogniti*") or ab ("Neurocogniti*") | 7589 |
|  | 8 | mh ("Neuropsychology" OR "Neuropsychological Tests") | 41131 |
|  | 9 | ti ("Cogniti* disorder*") or ab ("Cogniti* disorder*") or ti ("cogniti* defect*") or ab ("cogniti* defect*") or ti ("cogniti* deficit*") or ab ("cogniti* deficit*") or ti ("cogniti* disability*") or ab ("cogniti* disability*") or ti ("cogniti* dysfunction*") or ab ("cogniti* dysfunction*") or ti ("cogniti* impairment") or ab ("cogniti* impairment") or ti ("Cogniti* Decline*") or ab ("Cogniti* Decline*") or ti ("Mild Cognitive Impairment") or ab ("Mild Cognitive Impairment") or ti ("chemotherapy-related cognitive impairment") or ab ("chemotherapy-related cognitive impairment") or ti ("cancer related cognitive impairment") or ab ("cancer related cognitive impairment") | 19318 |
|  | 10 | mh ("Cognition Disorders" OR "Chemotherapy-Related Cognitive Impairment") | 36075 |
|  | 11 | (S1 OR S2) NOT (S3 OR S4) AND (S5 OR S6) AND (S7 OR S8) AND (S9 OR S10) | 24 |
|  | 12 | S11 AND (MH ("Cross Sectional Studies" OR "Correlational Studies" OR "Descriptive Research") OR TI ("cross section*" OR crosssection* OR crossection* OR correlational OR "descriptive stud*") OR AB ("cross section*" OR crosssection* OR crossection* OR correlational OR "descriptive stud*")) | **5** |
| 5)   **Scopus** | 1 | **TITLE-ABS (**Cancer* OR malignanc* OR neoplas* OR oncologi* OR tumo***)** | 4773224 |
|  | 2 | **TITLE-ABS (**"central nervous system neoplasm*" OR " central nervous system tumo*" OR " CNS tumo*" OR "Central Nervous System Cancer" OR " cns neoplasm*" OR "central nervous system neoplasms, primary" OR " neoplasm*, cns" OR "neoplasms, central nervous system" OR " primary central nervous system neoplasm*" OR " tumors, central nervous system "**)** | 9605 |
|  | 3 | **TITLE-ABS ("**Subjective OR complaint*" OR "subjective cognitive complaints" OR "subjective cognitive decline" OR "subjective memory complaints" OR "subjective memory decline" OR "perceived cognitive problems" OR "Report*, Self" OR "Self Report*" OR "Cognitive Symptom*"**)** | 303482 |
|  | 4 | **TITLE-ABS (**Neuropsycholog* OR "functioning of the brain and cognitive processes or behavior" OR "neuro-psychologi*" OR "Neurocogniti*") | **118020** |
|  | 5 | **TITLE-ABS ("**Cogniti* disorder*" OR "cogniti* defect*" OR "cogniti* deficit*" OR "cogniti* disability*" OR "cogniti* dysfunction*" OR "cogniti* impairment" OR "Cogniti* Decline*" OR "Mild Cognitive Impairment" OR "chemotherapy-related cognitive impairment" OR "cancer related cognitive impairment") | **157671** |
|  | 6 | #1 AND NOT #2 | **4763619** |
|  | 7 | #6 AND #3 AND #4 AND #5 | **116** |
|  | 8 | title-abs-key ("cross section*" OR crosssection* OR crossection*) | **1490936** |
|  | 9 | #7 AND #8 | **23** |
| 6) **Web of Science** | 1 | **ti=(** Cancer* OR malignanc* OR neoplas* OR oncologi* OR tumo***) or ab=(** Cancer* OR malignanc* OR neoplas* OR oncologi* OR tumo***)** | **4344437** |
|  | 2 | **ti=(**"central nervous system neoplasm*" OR " central nervous system tumo*" OR " CNS tumo*" OR "Central Nervous System Cancer" OR " cns neoplasm*" OR "central nervous system neoplasms, primary" OR " neoplasm*, cns" OR "neoplasms, central nervous system" OR " primary central nervous system neoplasm*" OR " tumors, central nervous system "**) or ab=(**"central nervous system neoplasm*" OR " central nervous system tumo*" OR " CNS tumo*" OR "Central Nervous System Cancer" OR " cns neoplasm*" OR "central nervous system neoplasms, primary" OR " neoplasm*, cns" OR "neoplasms, central nervous system" OR " primary central nervous system neoplasm*" OR " tumors, central nervous system "**)** | **7974** |
|  | 3 | **ti=(** **"**Subjective OR complaint*" OR "subjective cognitive complaints" OR "subjective cognitive decline" OR "subjective memory complaints" OR "subjective memory decline" OR "perceived cognitive problems" OR "Report*, Self" OR "Self Report*" OR "Cognitive Symptom*"**) or ab=("**Subjective OR complaint*" OR "subjective cognitive complaints" OR "subjective cognitive decline" OR "subjective memory complaints" OR "subjective memory decline" OR "perceived cognitive problems" OR "Report*, Self" OR "Self Report*" OR "Cognitive Symptom*"**)** | **271493** |
|  | 4 | **ti=(**Neuropsycholog* OR "functioning of the brain and cognitive processes or behavior" OR "neuro-psychologi*" OR "Neurocogniti*"**) or ab=(**Neuropsycholog* OR "functioning of the brain and cognitive processes or behavior" OR "neuro-psychologi*" OR "Neurocogniti*"**)** | **109222** |
|  | 5 | **ti=(** **"**Cogniti* disorder*" OR "cogniti* defect*" OR "cogniti* deficit*" OR "cogniti* disability*" OR "cogniti* dysfunction*" OR "cogniti* impairment" OR "Cogniti* Decline*" OR "Mild Cognitive Impairment" OR "chemotherapy-related cognitive impairment" OR "cancer related cognitive impairment"**) or ab=("**Cogniti* disorder*" OR "cogniti* defect*" OR "cogniti* deficit*" OR "cogniti* disability*" OR "cogniti* dysfunction*" OR "cogniti* impairment" OR "Cogniti* Decline*" OR "Mild Cognitive Impairment" OR "chemotherapy-related cognitive impairment" OR "cancer related cognitive impairment"**)** | **114675** |
|  | 6 | (#1) NOT (#2) AND (#3) AND (#4) AND (#5) | **115** |
|  | 7 | TS=("cross section*" OR crosssection* OR crossection*) | **981912** |
|  | 8 | **(#6) AND (#7)** | **9** |
